# Approaches to modeling treatment sequencing in practice: a thematic review of prior NICE appraisals

**DOI:** 10.1017/S0266462325103309

**Published:** 2025-11-27

**Authors:** Abualbishr Alshreef, Fern Woodhouse, Molly Haycock, Hugh Osborne, Dave Harland, Stephen Palmer

**Affiliations:** 1 AbbVie Inc, USA; 2 Costello Medical Consulting Ltd, UK; 3 AbbVie, New Zealand; 4 University of York, UK

**Keywords:** treatment sequencing, cost-effectiveness models, health technology assessment, decision making, health policy

## Abstract

**Background:**

As the variety of specific treatments in a disease area increases, there may be a growing interest in employing treatment sequencing within health economic models. The aim of this review was to identify and thematically analyze patterns regarding the approaches to modeling treatment sequencing in National Institute for Health and Care Excellence (NICE) appraisals.

**Methods:**

A review of NICE technology appraisals (TAs) published between 1 January 2020 and 13 March 2023 was conducted.

**Results:**

A total of twenty-four TAs incorporating treatment sequencing were included, most commonly in autoimmune and oncology indications. Primary justifications for companies employing treatment sequencing were precedence and alignment with clinical practice, whilst lack of appropriate clinical data was cited to justify its exclusion. Relatedly, External Assessment Groups commonly criticized treatment sequences for oversimplifying clinical practice. Notably, almost half of identified TAs assumed that the relative efficacy of an intervention was maintained regardless of disease severity or position within the treatment sequence.

**Conclusion:**

A substantial proportion of TAs employed treatment sequencing, but it is challenging to determine the impact of current approaches on the overall uncertainty associated with any health economic model. The challenges identified in this review could be used to inform future formal guidance and associated methodology for the implementation of treatment sequencing modeling, which could improve the comparability and reliability of models and their results.

## Introduction

In health technology assessment (HTA), health economic models are often required to evaluate the cost-effectiveness of new interventions versus currently available therapies. In such models, interventions are often individually assessed against comparators in a specific treatment line (i.e., distinct from the sequence in which they are received). However, the sequential receipt of interventions can be a key consideration for certain treatment pathways where the choice and effectiveness of subsequent treatments are influenced by previous treatments and patient characteristics (1).

As such, in some disease areas, namely immunological conditions where many interventions are available and patients cycle through several therapies, sequencing models have been used to reflect the order of treatments received and to facilitate the comparison of treatment sequences rather than treatments in isolation (2;3). In other areas such as oncology, treatment sequencing has also been shown to be prominent due to the transformations of treatment algorithms (i.e., the introduction of new innovative treatments) in recent years, which raise the need to tailor the sequence of different treatments to ensure the greatest possible efficacy (4). As the number of interventions available in a therapeutic area continues to increase over time, interest in employing treatment sequencing models may similarly continue to grow in the future (1–3;5).

Treatment sequencing models (i.e., for the purposes of this review, a model of at least two permutations of a sequence of discrete treatments accounting for both treatment effectiveness and cost) may also help to bridge the gap between typical evidence generation approaches (i.e., clinical trials assessing efficacy versus a few comparators in a defined treatment line) and real-world outcomes (3). However, there are challenges associated with treatment sequencing modeling; as previously highlighted by Lewis et al. (1) and Zheng et al. (3), randomized controlled trials of treatment sequences are scarce which necessitates the reliance on simplifying assumptions to bridge the data gap. As a consequence of these limitations, the use of treatment sequencing models has led to discord between companies and HTA bodies about how best to approach this type of modeling (6).

In a previous review of treatment sequencing models, Lewis et al. (1) highlighted that reviewing evidence of treatment sequencing is complex and challenging; treatment sequences often represent intricate, evolving intervention pathways that necessitate advanced quantitative evidence synthesis methods. Therefore, to contribute to the existing body of knowledge, this review aims to identify patterns regarding the utilization and perception of treatment sequencing from the perspective of the National Institute for Health and Care Excellence (NICE) in the United Kingdom (1;3;7). While Zheng et al. (3) investigated this research topic, their study was limited to appraisals published up to 2014. Consequently, this review aims to offer a more current perspective, focusing on NICE technology appraisals (TAs) published over a 3-year period between 2020 and 2023. Further, by thematically categorizing External Assessment Group (EAG)/NICE Committee critiques and recommendations, this review aims to provide a broad and novel perspective on treatment sequencing modeling; an area of keen interest to stakeholders seeking to optimize treatment sequencing strategies amidst an expanding number of treatment options.

## Methods

### Search strategy

The electronic search was carried out using the NICE website. NICE TAs published between 1 January 2020 and 13 March 2023 were reviewed using systematic methods. The search period was selected to identify the most recent TA precedence to supplement existing research (3;7), while ensuring a sufficient number of TAs that employed treatment sequencing modeling were included.

For the purposes of this review, treatment sequencing was defined as the modeling of at least two permutations of a sequence of discrete treatments accounting for both treatment effectiveness and cost (3;7;8). An example of what qualified as treatment sequencing is TA665, where the model evaluated treatment sequences of up to six treatments where the efficacy of each sequence was informed through a network meta-analysis. An example of what did not constitute as treatment sequencing were TAs that only included a bucket of subsequent treatment costs, or those that applied cost and/or efficacy inputs without considerations of the different permutations of treatment sequences.

### Inclusion and exclusion

TAs that met the eligibility criteria (Table 1) were added to an identification grid. Key terms were used as an initial check to identify relevant evaluations (Supplementary Table 1). While, to ensure a comprehensive search strategy, “treatment switching” was included as a key search term, it should be noted that TAs that *only* contained treatment switching methodology (outlined by NICE DSU TSD 16) (9) *without* specific consideration of the differences between treatment sequences in the cost-effectiveness model were not included.

**Table 1.** Eligibility criteria for the identification of relevant NICE TAs

Domain	Inclusion criteria	Exclusion criteria^a^
Timeframe	Date of NICE guidance publication from 1 January 2020 to 13 March 2023 (date of NICE website search)	Date of NICE guidance publication prior to 1 January 2020
Appraisal type/status	NICE Single Technology Appraisal NICE Multiple Technology Appraisal	NICE Cost Comparison^b^ Terminated NICE appraisal of any type CDF exit appraisals^c^
Modeling approach	Economic models that consider both treatment effects and costs of treatment sequences	Models that do not explicitly explore treatment sequencing

a Part reviews of previous full TAs were excluded following the development of the protocol as the data extracted may have fallen outside of the specified inclusion timeframe.

b Cost Comparison Appraisals (formerly referred to as Fast Track Appraisals) were excluded as their focus resides primarily in determining the relative cost of an intervention to a comparator, rendering the inclusion of treatment sequencing unlikely.

c CDF exit appraisals were excluded as the detail typically provided was considered insufficient to allow for the completion of the extraction grid. Additionally, due to their links to previous full TAs, any data extracted may have been obsolete relative to the specified inclusion timeframe.

*Abbreviations*: CDF, Cancer Drug Fund; NICE, National Institute for Health and Care Excellence; TA, technology appraisal.

For all TAs that could not be identified for inclusion by any of the key terms, the relevant, associated appraisal documents (Committee Papers, Appraisal Consultation Documents, Final Appraisal Documents) were manually reviewed for content pertaining to treatment sequencing by a single reviewer and potentially relevant TAs were discussed with a second reviewer to determine eligibility (ER, HO, respectively). If the two reviewers were unable to come to an agreement, a third independent reviewer made the final decision on inclusion of the TA (MH).

### Data extraction

Following identification of relevant TAs, data from associated appraisal documents were added to an extraction grid (Supplementary File 1). If treatment sequencing was implemented at any stage within the TA, full extraction was performed. If treatment sequencing was discussed but not modeled in the appraisal, data were only extracted for a subset of relevant categories within the extraction grid (herein termed “partially extracted”). For example, within TA718, the company’s economic model included the functionality to input costs and efficacy associated with subsequent lines of treatment, yet these fields were not completed due to an insufficient availability of evidence. Whilst this was highlighted as a source of uncertainty and discussed by both the Committee and the EAG, treatment sequencing was not implemented in any modeling analyses throughout the appraisal. This appraisal was therefore included within the identification stage but not fully extracted.

The review primarily focused on the methods for estimating clinical effectiveness of the treatment sequences, as well as the common challenges and criticisms raised around the approach taken for modeling treatment sequencing. Extracted details included information related to the TA itself, critiques from NICE and the EAG, company responses to critiques and final accepted approaches for decision making. In addition, the Final Scopes of fully extracted TA’s were retrospectively reviewed to confirm if treatment sequencing was mentioned throughout the NICE scoping stages.

Data extraction was performed by a single reviewer for each included TA (ER and HO). A second reviewer independently verified the extracted information (MH and FW). The extracted qualitative information was thematically analyzed to identify key approaches and challenges for modeling treatment sequencing.

## Results

### Characteristics of extracted studies

**Figure 1.**
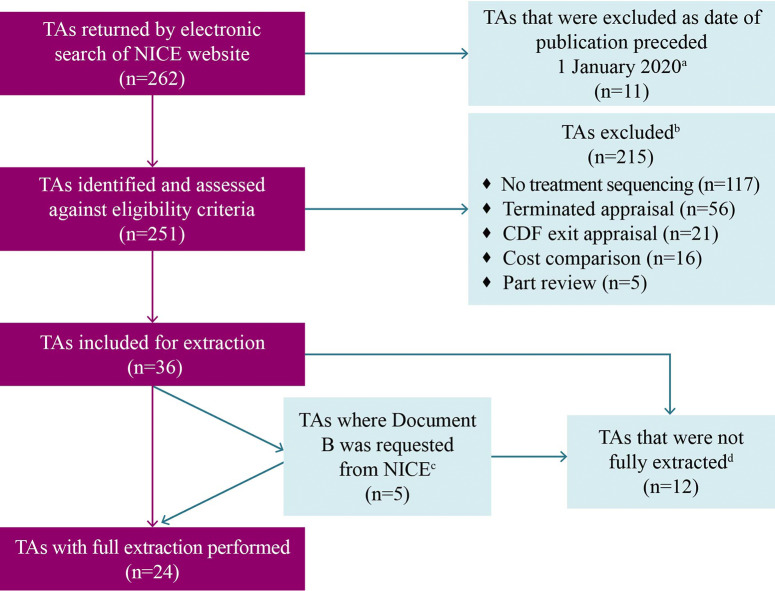
PRISMA flow diagram of the TA identification and extraction workflow. ^a^Appraisals with an original date of publication preceding 1 January 2020 were returned in the electronic search of the NICE website due to updates in associated NICE guidance published after 1 January 2020. ^b^TAs may have met multiple exclusion criteria, but only one criterion is specified for each TA. ^c^If the published Committee Papers for the appraisal only included Document A (a summary document), Document B (the full document) was requested from NICE during the Appraisal Extraction stage. ^d^Appraisals that were identified but partially extracted included those that contained only a mention of treatment sequencing or those whose modeling when reviewed in detail did not constitute treatment sequencing. A partial extraction consisted of extracting all available information in the appraisal relevant to treatment sequencing. *Abbreviations*: CDF, Cancer Drugs Fund; NICE, National Institute for Health and Care Excellence; PRISMA, Preferred Reporting Items for Systematic reviews and Meta-Analyses; TA, technology appraisal.

A total of 262 TAs were returned by the electronic search, of which 251 were published within the specified date range. Of the total TAs identified, thirty-six (36/251; 14.3 percent) fulfilled the eligibility criteria for further extraction (Supplementary Table 2), these included both single TAs (*n* = 34) and multiple TAs (*n* = 2). Overall, twenty-four TAs (24/251; 9.6 percent) were fully extracted, and twelve TAs were partially extracted (12/251; 4.8 percent) (Figure 1).

**Figure 2.**
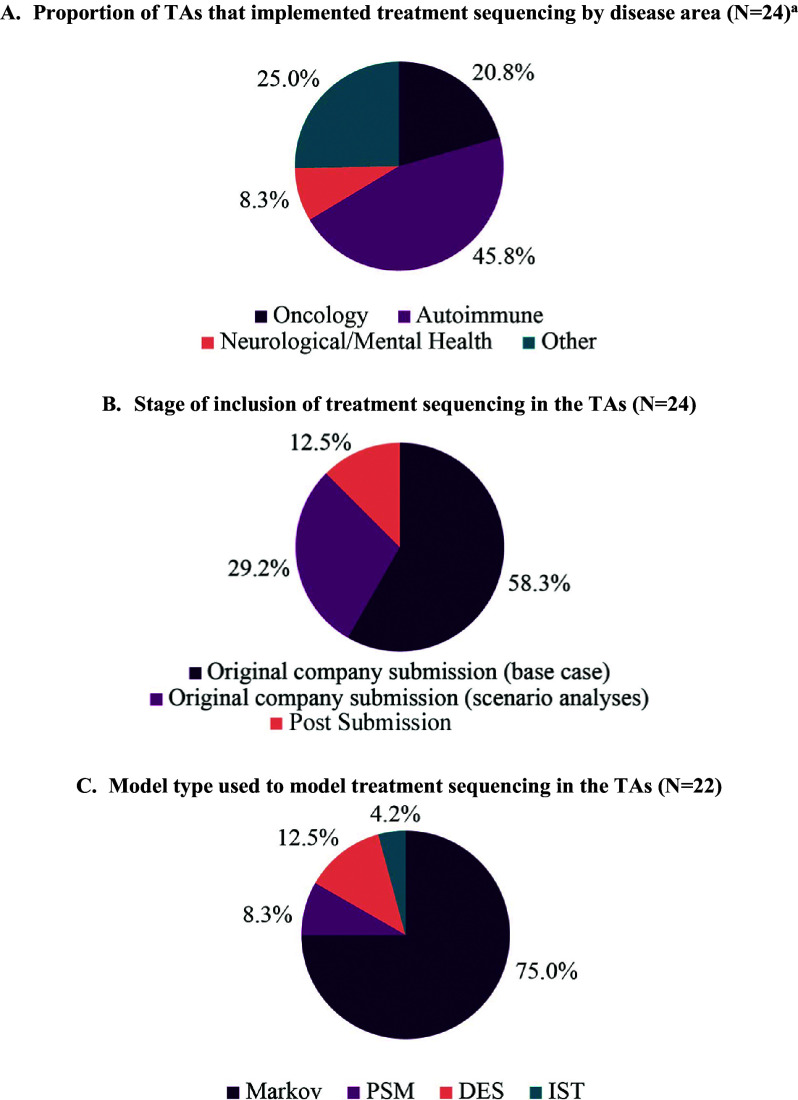
(A–C) Pie charts of results. “Other” indications included atopic dermatitis, eosinophilic esophagitis, human immunodeficiency virus 1, thrombocytopenia and osteoporosis. *Abbreviations*: DES, discrete event simulation; IST, individual state transition; PSM, partitioned survival model; TA, technology appraisal.

Of the TAs that formally evaluated treatment sequencing, interventions in autoimmune indications were most common (11/24; 45.8 percent; Figure 2A), featuring particularly in ulcerative colitis, rheumatoid arthritis and axial spondyloarthritis. TAs submitted in oncology indications were the second most frequent when excluding the broad “other” category (5/24; 20.8 percent; Figure 2A). Three of the five oncology TAs (3/5; 60.0 percent) involved interventions in prostate cancer (metastatic and non-metastatic).

**Figure 3.**
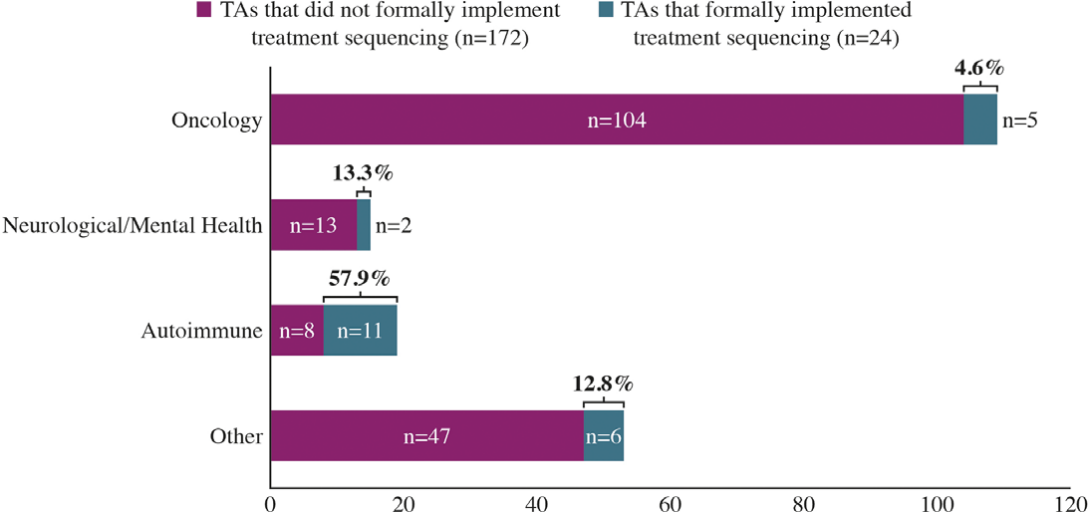
Proportion of non-terminated TAs that implemented treatment sequencing by disease area (*N* = 196). “Other” indications include, but are not limited to, cardiovascular disease, diabetes, atopic dermatitis, eosinophilic esophagitis, human immunodeficiency virus 1, thrombocytopenia and osteoporosis. *Abbreviation*: TA, technology appraisal.

Relative to the high number of non-terminated TAs submitted in oncology indications (109/196; 55.6 percent), treatment sequencing was infrequently implemented (5/109; 4.6 percent), compared with TAs in autoimmune indications (11/19; 57.9 percent: Figure 3).

Overall, 196 identified TAs (196/251; 78.1 percent) were not terminated. Among the extracted TAs that implemented treatment sequencing, NICE recommended these at a similar rate (22/24; 91.7 percent) to TAs which did not formally assess treatment sequences (162/172; 94.2 percent).

### Nature of inclusion of treatment sequencing

Primary justifications for companies implementing treatment sequencing modeling were precedence within disease areas and alignment with clinical practice. Treatment sequencing was not mentioned in the Final Scopes for any of the TAs that formally evaluated treatment sequencing. Treatment sequencing was most frequently incorporated in a model at the initial company submission stage of the TA in the base case analysis (14/24; 58.3 percent; Figure 2B). In 29.2 percent (7/24) of the TAs, treatment sequencing featured as part of one or multiple scenario analyses, rather than the base case analysis. In TAs that did not include treatment sequencing in the initial company submission, treatment sequencing was occasionally included in scenario analyses following the EAG report (3/24; 12.5 percent).

Treatment sequencing was mentioned first by the EAG in three of the identified TAs (3/24; 12.5 percent), but was ultimately not implemented as the Committee deemed the company submissions sufficiently representative of clinical practice. In TA853 as an example, this decision was rooted in clinical expert opinion validating that treatment for the disease is highly individualized due to its variability, resulting in no “fixed” treatment sequence in clinical practice; this supported the adequacy of the company’s ingoing mixed treatment approach.

In 75 percent (9/12) of partially extracted TAs, treatment sequencing was explicitly mentioned during the appraisal but not implemented. On five occasions, treatment sequencing was acknowledged by the company as a suitable approach but concluded to be unfeasible due to a paucity of clinical data (5/9; 55.6 percent).

### Modeling approaches

Treatment sequencing was most often modeled as part of a Markov model (Figure 2C). All TAs that formally included treatment sequencing reported results as an incremental cost-effectiveness ratio (ICER) using a fully incremental approach. In one appraisal (TA828), net health benefit (NHB) values were also presented.

While the maximum number of treatment lines in a given sequence was found to be as high as eight, the majority of TAs evaluated a smaller number of treatment lines (three to five). Treatment sequences were sometimes repeated across patient subpopulations according to disease severity and/or treatment resistance. This resulted in the number of individual treatment sequences considered being as high as 71 (e.g., TA665, TA744). However, the number of sequences presented was typically smaller, particularly for interventions in non-autoimmune indications. As the sequences presented may not have captured the totality of possible treatment sequences, restrictions in the sequences conducted were frequently justified using clinical expert opinion, market share data and alignment with previous TAs in the same indication.

In some instances, treatment sequencing was only modeled in a certain disease stage (e.g., progressed disease), using subhealth states to represent different lines of treatment. This most often occurred in oncology indications, likely due to the prevalence of defined health states in this disease area (e.g., metastasis, progressed disease) and the potential impact of subsequent treatment lines on costs and outcomes. Similar findings were observed for transition probabilities and subsequent treatment line switches, which were typically informed by disease progression or the time-to-discontinuation data from relevant clinical trials and/or clinical validation.

Another approach, most often observed in autoimmune indications, involved companies reflecting the principal clinical trial design through modeling an initial double-blinded, placebo-controlled trial period via a series of tunnel states until the time at which the primary endpoint was evaluated. This was then followed by a maintenance or continuous treatment period in which patients transitioned between subhealth states associated with different maintenance treatments. While less common, a number of autoimmune TAs also incorporated adverse event data from relevant clinical trials to inform transition probabilities for changing treatment (2/11; 18.2 percent). Additionally, data informing the likelihood of switching treatments were commonly stratified according to patient subgroups, determined by prior exposure to a given drug class or disease severity, and were informed by the associated patient subgroup’s clinical effectiveness data in the trial(s) (10/11; 90.9 percent).

### Model assumptions

There was variation in the model assumptions adopted across the TAs reviewed. In the absence of suitable efficacy data specific to line of therapy, almost half (11/24; 45.8 percent) of TAs employed the assumption that the relative efficacy of a given intervention was maintained regardless of disease severity or position within the treatment sequence. When available, efficacy data specific to an intervention being used in a particular line of therapy were used; broadly, treatment effectiveness data specific to an intervention being used as a second- or third-line therapy were more commonly available in autoimmune disease indications (7/11; 63.6 percent). Some TAs (4/24; 16.7 percent) also applied a similar assumption to discontinuation rates, which were frequently assumed by the company to remain constant over the model horizon irrespective of position within a treatment sequence.

In some models, an initial trial period was modeled directly until the time at which the primary endpoint was evaluated to reflect the data of the principal clinical trial(s); rates of treatment discontinuation were sometimes not explicitly modeled in these tunnel states (4/24; 16.7 percent). Equally, following discontinuation of the last line of active therapy, it was often assumed that patients would remain on best supportive care until the end of the time horizon.

Adjustment of the measured treatment effect was also observed and was more prevalent in autoimmune TAs compared with other indications. This assumption was most commonly implemented via waning of treatment effect whilst off-treatment, as well as one example of treatment effect waning whilst on-treatment (TA828). Moreover, the impact of possible effect degradation by treatment line was explored in two TAs (TA854 and TA719), with the latter TA assuming the same decrement across all active treatments.

### Critiques and preferences of company approaches to treatment sequencing

#### EAG critiques

EAGs frequently criticized treatment sequences for being an oversimplification of clinical practice and the potential impact of this on cost-effectiveness results (Table 2). Additionally, it was occasionally noted that extended sequences, in combination with the assumption of equal relative efficacy, inherently benefitted longer treatment sequence strategies when assessing cost-effectiveness. In these cases, the EAG suggested that the treatment sequences should align more closely to the treatment pathway in clinical practice, by modeling additional treatment sequences and/or further treatment lines. To address this, in six appraisals, EAGs remodeled the base case or performed their own modeling of additional treatment sequences. Occasionally, the EAG suggested that the company perform additional scenario analyses to explore the cost-effectiveness of a given treatment sequence in a patient subpopulation, such as disease severity subpopulations (e.g., TA676).

**Table 2.** Frequent EAG critiques of treatment sequencing modeling

EAG critique	Number of relevant appraisals	Appraisals recommended
Treatment sequences represent an oversimplification of clinical practice^a^	17/24 (70.8%)	16/17 (94.1%)
Invalid justification for treatment sequences^b^	13/24 (54.2%)	13/13 (100%)
Inconsistency of treatment sequencing approach with prior TAs in the same or similar indication	4/24 (16.7%)	4/4 (100%)

a With respect to the treatments available or disease progression, and that the company submission did not explore the fullest range of possible sequences.

b If a fixed number of treatment sequences and permutations was explored.

*Abbreviations*: EAG, External Assessment Group; TA, technology appraisal.

In 29.2 percent (7/24) of TAs, the companies reinforced their original arguments (e.g., lack of suitable clinical data, alignment with sequence precedence in the disease area, and reaffirming the companies modeling as reflective of clinical practice) in response to EAG critiques without making changes to their modeling approach. These TAs were subsequently all recommended. In the remaining TAs (17/24; 70.8 percent), companies either part-implemented or fully implemented the EAG’s requests. Of these TAs, 88.2 percent (15/17) were subsequently recommended.

#### Committee preferences and recommendations

Across TAs that implemented (fully extracted TAs) or discussed treatment sequencing (partially extracted TAs), minor changes to the inclusion of treatment sequencing were sometimes requested by the Committee (2/36; 5.6 percent). Across fully extracted TAs Committee critiques often centered around the treatment sequences not being entirely reflective of clinical practice (9/24; 37.5 percent), where the resulting recommendation was to implement the sequences suggested by the EAG. Model assumptions were also critiqued by the Committee (4/24; 16.7 percent), such as when the time-on-treatment or treatment distributions at various treatment lines were considered unrealistic.

The company base case approach to modeling treatment sequencing as submitted was accepted for decision making in a minority of cases (6/24; 25.0 percent); with the Committee also considering both the company base case and the revised EAG base case in parallel in two of these cases (8.3 percent). In most occasions, the base case approach to treatment sequencing was ultimately accepted for decision making following the implementation of EAG and/or Committee recommendations (16/24; 66.7 percent). The remaining TAs were not recommended (2/24, 8.3 percent).

## Discussion

A notable proportion of all TAs published between January 2020 and March 2023 discussed treatment sequencing modeling approaches. Even if incorporation of treatment sequencing may increase complexity and add additional uncertainty, this review indicates that there is no evidence that the incorporation of treatment sequencing negatively impacts the overall rate of reimbursement. However, this review did not consider whether other factors of reimbursement (such as the time to final decision) are impacted by inclusion of treatment sequencing modeling.

Precedence was frequently cited as justification for inclusion of treatment sequencing modeling, which may explain why it was most frequently modeled at the initial company submission and rarely thereafter. It is possible that “seeding” of these modeling approaches in an indication promotes subsequent uptake by other companies. This was particularly apparent in certain disease areas, namely autoimmune conditions, as well as within specific indications, such as ulcerative colitis and prostate cancer. This observation may also be explained by certain disease areas being better suited to the use of treatment sequencing due to how they are managed in clinical practice. Notably, autoimmune indications, where switching between therapies is prevalent, modelled treatment sequencing more often. Additionally, practical difficulties associated with structurally adapting a model *post hoc* to incorporate treatment sequencing approaches may further explain why they were rarely incorporated after the original company submission. Consequently, it may be important for companies to consider treatment sequencing early in the model development process to ensure models are built with the relevant capacity to incorporate treatment sequencing, if deemed appropriate. In terms of the frequency of adoption of treatment sequencing approaches, the proportion of TAs published between January 2020 and March 2023 discussing treatment sequencing modeling approaches identified in this review was lower than that reported by Zheng et al. (3), who reviewed all NICE TAs published up to 2014 (9.6 percent versus 16.1 percent). In addition, the proportion of oncology TAs identified in this review that included treatment sequencing was lower than that reported by Huang et al. (7), who reviewed oncology NICE TAs published between 2014 and 2019 (3.6 percent versus 16.0 percent).

While variation in the assumptions adopted in TAs was observed, findings on model assumptions in this review broadly align with previous published findings; this indicates limited advancement has taken place in this field since previous research was conducted. In particular, the assumption of equal relative efficacy of treatments regardless of position in the treatment sequence was still frequently observed across the appraisals reviewed, which aligns with the prior literature (1;7). Such an assumption may be necessary in the absence of tailored evidence generation projects, particularly for treatment effectiveness data beyond second line, where calculating more accurate cost-effectiveness estimates would require treatment line-specific clinical data. Additionally, it should be caveated that various factors contribute to the most appropriate modeling approach; therefore further research (such as that conducted previously) (1;7;10) investigating the correlation between the source data and treatment sequencing statistical approaches could be warranted.

Moreover, as a result of the strong assumptions often used (along with the comparison of treatment sequences with varying lengths) meaningful interpretation of cost-effectiveness results based on treatment sequencing models can be difficult, especially when the difference in treatment effect between the intervention and its comparators is small. The inherent limitations of the ICER as a gauge for relative cost-effectiveness (namely its lack of sensitivity to small differences in treatment effect) may contribute to these challenges (11;12). With this considered, it may not be appropriate to rank the cost-effectiveness of treatment sequences according to their respective ICERs, and absolute measures of cost-effectiveness, such as NHB, may be better suited (provided that the willingness-to-pay threshold is known) (11–13).

Related to the lack of tailored evidence generation projects, companies often defended their modeling approach based on a lack of suitable clinical data, nonetheless, EAGs frequently determined the approach as an oversimplification of clinical practice. This may be because the number and length of sequences modeled were a function of the data available, rather than alignment with clinical practice. This hypothesis is supported by Zheng et al. (3), who noted that the assumption of equal relative efficacy regardless of sequence position was necessary in the absence of suitable clinical data to inform these further modeled treatment lines. This is further supported by Lewis et al. (1), who added that randomized controlled trials in their current format are limited with regards to their use in treatment sequencing models, and models are constrained by the head-to-head comparisons of discrete treatments that are often the basis of such studies (1;3). As a result, the handling of this paucity in clinical data is dealt with during the modeling phase rather than by addressing evidence generation methodology (1;3).

To improve treatment sequencing modeling, it might be necessary to tailor evidence generation projects to provide relevant data, including for comparators and subsequent treatments, to permit modeling which is more reflective of clinical practice, as outlined by Lewis et al. (1) and Diao et al. (14). However, such evidence generation projects are associated with obstacles; they are resource-intensive and recruiting large patient cohorts when patients are randomized to treatments with differing efficacy for several treatment lines can be ethically challenging (15). Further, such trials may quickly become outdated as new therapies enter the market (15). Therefore, companies need to balance these limitations with the degree to which treatment sequencing modeling improves health economic models when such data are available, especially considering that the current approaches outlined in this review do not appear to hinder reimbursement.

When considering the simplifying assumptions taxonomy developed by Lewis et al. (1), not all assumptions were identified in this review, namely “displacement effect ignored” and “the use of uncontrolled/observational studies without bias adjustment.” The majority of TAs modeled several treatment lines within a given sequence and assumed patients would remain on best supportive care for the remainder of the time horizon, following discontinuation of the last line of active therapy. This observation may contrast with alternative methods, such as those proposed by van de Wetering et al. (8), where all subsequent lines of treatment are combined into a single basket before switching to best supportive care.

The lack of formal guidance for treatment sequencing modeling from NICE or the Decision Support Unit (DSU), combined with the infrequent evaluation of such models, may intensify the dependence on precedence and the need for Committees to accept strong assumptions. While NICE are considering treatment sequencing modeling as part of their proportionate approach to TAs, such as the Renal Cell Carcinoma Pathways Pilot (ID6186) (16), it is challenging to ascertain whether the complexity associated with treatment sequencing modeling, and the influence this may have on uncertainty, is outweighed by an improved alignment with clinical practice (17). Nonetheless, the recent recommendation of TA964 (cabozantinib with nivolumab for untreated advanced renal cell carcinoma) based on the Renal Cell Carcinoma Pathways Pilot, indicates a potential move toward increased usage of treatment sequencing modeling in HTA submissions in the future.

### Limitations

This review delivers a contemporary and extensive viewpoint on treatment sequencing modeling; an area of great interest to stakeholders striving to enhance treatment sequencing strategies in the context of a growing variety of treatment options. Nonetheless, as the search criteria for this review were set up to identify TAs where treatment sequencing was discussed, the review did not identify TAs in which treatment sequencing may have been appropriate, but not discussed; further investigation into this may be warranted. Furthermore, highly specialized TAs (HSTs) were not included within the scope of this review. Regardless, the impact of this limitation is expected to be minimal, as the likelihood of HSTs utilizing treatment sequencing is low, given that these are typically innovative technologies for rare conditions where there are currently very limited treatment options available.

Moreover, although NICE decision making is well-respected internationally and trends in NICE decision making may impact HTA decision making in other markets, the results identified in this review are specific to the United Kingdom; further research would be required to investigate the acceptance of treatment sequencing by global HTA bodies.

Finally, although the timeframe of extraction within this review was selected to ensure more recent treatment sequencing methods and trends were captured, TAs published earlier than 2020 were not reviewed. Further, there is scope for future research to explore TAs published between 2023 and the present.

### Recommendations

Industry-wide, companies should facilitate generation and reporting of the necessary efficacy and cost data for sequencing to be modeled and utilized for HTA processes. Further, the appropriateness of treatment sequencing should be considered early in the model development process to ensure models have the necessary capacity and accurately reflect clinical practice.

HTA agencies and associated bodies, such as NICE and its DSU, should be encouraged to develop clarity on treatment sequencing modeling, whether that be through continuing to consider treatment sequencing modeling as part of their proportionate approach to TAs, or through formal guidance. In addition, to ensure early alignment on modeling approaches, NICE TAs should identify the need to evaluate treatment sequences in the initial scope.

## Conclusions

Overall, a notable proportion of TAs employed treatment sequencing within the timeframe set for this review. However, it is challenging to determine the degree to which current treatment sequencing approaches impact the overall uncertainties associated with health economic models. Nevertheless, the key challenges and critiques identified in this review, such as the need for companies to rely on simplifying assumptions due to paucity of data, reaffirm findings from previous literature from a more current perspective. These insights can be used to inform future research and help develop a methodological framework for the implementation of treatment sequencing modeling.

While a fully generalizable approach may not be attainable, and further research is warranted into the use and standardized implementation of treatment sequencing modeling, methodological alignment across disease areas and/or indications could greatly enhance the comparability and reliability of models and the results obtained. Therefore, companies should encourage and support NICE and the DSU in providing clarity on treatment sequencing to ensure best practices are clearly established.

## Supporting information

Alshreef et al. supplementary material 1 Alshreef et al. supplementary material

Alshreef et al. supplementary material 2 Alshreef et al. supplementary material

## Supplementary material

The supplementary material for this article can be found at http://doi.org/10.1017/S0266462325103309.
