# Supplementary material for: Approaches to modeling treatment sequencing in practice: a thematic review of prior NICE appraisals
Source: Int J Technol Assess Health Care. 2025 Nov 27;41(1):e88. doi: 10.1017/S0266462325103309 (PMC12723307; doi:10.1017/S0266462325103309)
Supplement: Alshreef et al. supplementary material 1 — Alshreef et al. supplementary material [file S0266462325103309sup001.docx]

# SUPPLEMENTARY INFORMATION

Supplementary Table 1. Key search terms included in the identification process

| **Number** | **Terms** |
| --- | --- |
| 1 | Treatment sequenc* |
| 2 | Treatment-sequenc* |
| 3 | Sequen* |
| 4 | Treatment pattern* |
| 5 | Treatment switch* |
| 6 | Switch* |
| 7 | Failure |
| 8 | Naïve |
| 9 | Experienced |
| 10 | Exposed |

**Footnotes: ‘***’ indicates that all forms of the word following its inclusion were flagged by a text-based search, similarly to a Boolean wildcard function.

Supplementary Table 2: Overview of extracted TAs (N=36)

| Appraisal title | ID | Date | | Company details | EAG details | Appraisal type | Disease area | Base case or scenario analysis | Outcome |
| --- | --- | --- | --- | --- | --- | --- | --- | --- | --- |
| Full extraction (N=24) | | | | | | | | | |
| Ustekinumab for treating moderately to severely active ulcerative colitis | TA633 | 17/06/2020 | | Janssen | Southampton Health Technology Assessments Centre | STA | Autoimmune | Scenario analysis | Recommended^a^ |
| Darolutamide with androgen deprivation therapy for treating hormone-relapsed non-metastatic prostate cancer | TA660 | 25/11/2020 | | Bayer | Aberdeen HTA Group | STA | Oncology | Base case | Recommended^b^ |
| Upadacitinib for treating severe rheumatoid arthritis | TA665 | 09/12/2020 | | AbbVie | Peninsula Technology Assessment Group | STA | Autoimmune | Base case | Recommended^a^ |
| Filgotinib for treating moderate to severe rheumatoid arthritis | TA676 | 24/02/2021 | | Gilead Sciences | Kleijnen Systematic Reviews Ltd | STA | Autoimmune | Base case | Recommended^a^ |
| Baricitinib for treating moderate to severe atopic dermatitis | TA681 | 03/03/2021 | | Eli Lilly | CRD and CHE Technology Assessment Group, University of York | STA | Other | Scenario analysis | Recommended^a^ |
| Anakinra for treating Still’s disease | TA685 | 31/03/2021 | | Swedish Orphan Biovitrum | Liverpool Reviews & Implementation Group | STA | Autoimmune | Base case | Recommended^a^ |
| Budesonide orodispersible tablet for inducing remission of eosinophilic esophagitis | TA708 | 23/06/2021 | | Dr Falk Pharma UK Ltd | Southampton HTA centre | STA | Other | Scenario analysis | Recommended^a^ |
| Enzalutamide for treating hormone-sensitive metastatic prostate cancer | TA712 | 07/07/2021 | | Astellas | University of Aberdeen HTA Group | STA | Oncology | Base case | Recommended^a^ |
| Secukinumab for treating non-radiographic axial spondylarthritis | TA719 | 21/07/2021 | | Novartis | CRD and CHE Technology Assessment Group, University of York | STA | Autoimmune | Scenario analysis | Recommended^c^ |
| Abiraterone for treating newly diagnosed high-risk hormone-sensitive metastatic prostate cancer | TA721 | 18/08/2021 | | Janssen | Aberdeen HTA Group | STA | Oncology | Scenario analysis | Not recommended |
| Upadacitinib for treating moderate rheumatoid arthritis | TA744 | 10/11/2021 | | AbbVie | Peninsula Technology Assessment Group | STA | Autoimmune | Base case | Recommended^a^ |
| Cenobamate for treating focal onset seizures in epilepsy | TA753 | 15/12/2021 | | Arvelle Therapeutics | CRD and CHE Technology Assessment Group, University of York | STA | Neurology/mental health | Base case | Recommended^a^ |
| Cabotegravir with rilpivirine for treating HIV-1 | TA757 | 05/01/2022 | | ViiV Healthcare | Kleijnen Systematic Reviews | STA | Other | Base case | Recommended^a^ |
| Osimertinib for adjuvant treatment of EGFR mutation-positive non-small-cell lung cancer after complete tumor resection | TA761 | 19/01/2022 | | AstraZeneca | School of Health and Related Research | STA | Oncology | Base case | Recommended^a^ |
| Upadacitinib for treating active psoriatic arthritis after inadequate response to DMARDs | TA768 | 02/02/2022 | | AbbVie | Liverpool Reviews and Implementation Group | STA | Autoimmune | Base case | Recommended^b^ |
| Romosozumab for treating severe osteoporosis | TA791 | 25/05/2022 | | Union Chimique Belge Pharma | Kleijnen Systematic Reviews | STA | Other | Base case | Recommended^a^ |
| Filgotinib for treating moderately to severely active ulcerative colitis | TA792 | 01/06/2022 | | Galapagos | Kleijnen Systematic Reviews | STA | Autoimmune | Scenario analysis | Recommended^c^ |
| Abrocitinib, tralokinumab or upadacitinib for treating moderate to severe atopic dermatitis | TA814 | 03/08/2022 | | Pfizer (abrocitinib), Leo Pharma (tralokinumab) and AbbVie (upadacitinib) | British Medical Journal Technology Assessment Group | MTA | Other | Scenario analysis | Recommended^a^ |
| Guselkumab for treating active psoriatic arthritis after inadequate response to DMARDs | TA815/TA711 | 10/08/2022 | | Janssen | CRD, University of York | STA | Autoimmune | Base case | Recommended^a^ |
| Ozanimod for treating moderately to severely active ulcerative colitis | TA828 | 05/10/2022 | | Celgene Ltd | Peninsula Technology Assessment Group | STA | Autoimmune | Scenario analysis | Recommended^c^ |
| Pembrolizumab for adjuvant treatment of resected stage 2B or 2C melanoma | TA837 | 26/10/2022 | | Merck Sharp & Dohme | Kleijnen Systematic Reviews | STA | Oncology | Base case | Recommended^a^ |
| Avatrombopag for treating primary chronic immune thrombocytopenia | TA853 | 15/12/2022 | | Swedish orphan biovitrum | University of York | STA | Other | Scenario analysis (EAG) | Recommended^c^ |
| Esketamine nasal spray for treatment-resistant depression | TA854 | 14/12/2022 | | Janssen | Kleijnen Systematic Reviews | STA | Neurology/mental health | Base case | Not recommended |
| Upadacitinib for treating moderately to severely active ulcerative colitis | TA856 | 04/01/2023 | | AbbVie | Liverpool Reviews & Implementation Group | STA | Autoimmune | Scenario analysis | Recommended^a^ |
| Partially extracted (N=12)^b^ | | |  |  |  |  |  |  |  |
| Peginterferon beta-1a for treating relapsing–remitting multiple sclerosis | TA624 | 19/02/2020 | | Biogen | Warwick Evidence | STA | Neurology/mental health | – | Recommended |
| Ozanimod for treating relapsing–remitting multiple sclerosis | TA706 | 09/06/2021 | | Celgene UK Ltd/ Bristol-Myers Squibb | Liverpool Reviews & Implementation Group | STA | Neurology/mental health | – | Not recommended |
| Ravulizumab for treating atypical hemolytic uremic syndrome | TA710 | 23/06/2021 | | Alexion Pharma | CRD, University of York | STA | Cardiovascular | – | Recommended |
| Ixekizumab for treating axial spondylarthritis | TA718 | 21/07/2021 | | Eli Lilly | Liverpool Reviews and Implementation Group | STA | Autoimmune | – | Recommended |
| Chlormethine gel for treating mycosis fungoides-type cutaneous T-cell lymphoma | TA720 | 18/08/2021 | | Recordati Rare Diseases/Helsinn | University of Aberdeen HTA Group | STA | Oncology | – | Recommended |
| Apalutamide with androgen deprivation therapy for treating high-risk hormone-relapsed non-metastatic prostate cancer | TA740 | 28/10/2021 | | Janssen | Southampton HTA centre | STA | Oncology | – | Recommended |
| Apalutamide with androgen deprivation therapy for treating hormone-sensitive metastatic prostate cancer | TA741 | 28/10/2021 | | Janssen | Southampton HTA centre | STA | Oncology | – | Recommended |
| Solriamfetol for treating excessive daytime sleepiness caused by narcolepsy | TA758 | 05/01/2022 | | Jazz Pharmaceuticals | Southampton Health Technology Assessments Centre | STA | Neurology/mental health | – | Recommended |
| Ponesimod for treating relapsing–remitting multiple sclerosis | TA767 | 02/02/2022 | | Janssen | Peninsula Technology Assessment Group | STA | Neurology/mental health | – | Recommended |
| Venetoclax with low dose cytarabine for untreated acute myeloid leukemia when intensive chemotherapy is unsuitable | TA787 | 27/04/2022 | | AbbVie | Aberdeen HTA Group | STA | Oncology | – | Recommended |
| Nivolumab for adjuvant treatment of invasive urothelial cancer at high risk of recurrence | TA817 | 10/08/2022 | | Bristol-Myers Squibb | School of Health and Related Research | STA | Oncology | – | Recommended |
| Lenvatinib with pembrolizumab for untreated advanced renal cell carcinoma | TA858 | 11/01/2023 | | Eisai; Merck Sharp & Dohme | Liverpool Reviews and Implementation Group | MTA | Oncology | – | Recommended |

**Footnotes:** ^a^Approach to modeling treatment sequencing was only accepted for decision-making following the implementation of EAG and/or Committee recommendations. ^b^Committee considered both the Company and the EAG revised treatment sequencing base case in parallel for decision-making. ^c^Ingoing Company approach to modeling treatment sequencing (as submitted) was accepted for decision-making. ^d^Appraisals that were identified but partially extracted included those that contained only a mention of treatment sequencing or those whose modeling when reviewed in detail did not constitute treatment sequencing.

**Abbreviations:** CHE: Centre for Health Economics; CRD: Centre for Reviews and Dissemination; DMARD: disease-modifying anti-rheumatic drug; EAG: External Assessment Group; EGFR: epidermal growth factor receptor; HIV-1: Human Immunodeficiency Virus-1; ID: identification number; MTA: multiple technology appraisal; STA: single technology appraisal; TA: technology appraisal.
