# Supplementary material for: Approaches to modeling treatment sequencing in practice: a thematic review of prior NICE appraisals
Source: Int J Technol Assess Health Care. 2025 Nov 27;41(1):e88. doi: 10.1017/S0266462325103309 (PMC12723307; doi:10.1017/S0266462325103309)
Supplement: Alshreef et al. supplementary material 2 — Alshreef et al. supplementary material [file S0266462325103309sup002.docx]

**Supplementary Materials**

**Supplementary Table 1:** Identification Grid

| **Appraisal Details** | | | | | | **Indication** | |
| --- | --- | --- | --- | --- | --- | --- | --- |
| **Appraisal title** | **Appraisal ID** | **Appraisal date** | **Appraisal type** | **Outcome** | **Link to NICE technology appraisal guidance** | **Indication** | **Disease area** |
| Appraisal title | TAXXX | 3/10/2023 | Single technology appraisal | Recommended | URL | Indication | Oncology |
|  |  |  |  |  |  |  |  |

**Supplementary Table 1:** Identification Grid (continued)

| **Intervention** | | | **Key Term Identified** | **Manual Check** | **Exclusion** | **Final Inclusion Decision** |
| --- | --- | --- | --- | --- | --- | --- |
| **Drug name** | **Drug class** | **Mechanism of action** | **Key term identified from Table 1 (#) of Protocol** | **Rationale for inclusion** | **Rationale for exclusion** | **To be fully extracted? (Y/N)** |
| Name | Class | MoA | 6 | NICE STA and treatment sequencing model | Not applicable | Y |
|  |  |  |  |  |  |  |

**Supplementary Table 2:** Extraction Grid

| **Appraisal Details** | | | | | | **Indication** | |
| --- | --- | --- | --- | --- | --- | --- | --- |
| **Appraisal title** | **Appraisal ID** | **Appraisal date** | **Company details** | **Committee details** | **External Assessment Group (EAG) details** | **Indication** | **Disease area** |
| Appraisal title | TAXXX | 3/10/2023 | Pharmaceutical Company | Committee A | EAG | Indication | Oncology |
|  |  |  |  |  |  |  |  |

**Supplementary Table 2:** Extraction Grid (continued)

| **Intervention** | | | **Treatment Sequence Modelling** | | | | | |
| --- | --- | --- | --- | --- | --- | --- | --- | --- |
| **Drug name** | **Drug class** | **Mechanism of action** | **Company approach to modelling** | **Model type** | **Input data source and type** | **Number of sequence permutations explored and associated rationale** | **Reporting of cost-effectiveness results** | **Model assumptions** |
| Name | Class | MoA | Approach to modelling in original submission Revised approach to modelling during technical engagement | Cohort state-transition | Input data source and type | Number of sequence permutations explored and associated rationale | ICER | Model assumptions |
|  |  |  |  |  |  |  |  |  |

**Supplementary Table 2:** Extraction Grid (continued)

| **Relevant External Input** | | | | |
| --- | --- | --- | --- | --- |
| **EAG critique** | **EAG recommendations** | **Company’s response during post-submission stage, and details of resulting new models (if any)** | **Appraisal committee critique** | **Appraisal committee recommendations** |
| EAG critique | EAG recommendations | CQs: Company response; TE: Company response; ACD: Company response | Appraisal committee critique | Appraisal committee recommendations |
|  |  |  |  |  |

**Supplementary Table 2:** Extraction Grid (continued)

| **Nature of Inclusion of Treatment Sequencing** | | | **Outcome** | |
| --- | --- | --- | --- | --- |
| **Original submission** | **Following committee meeting (#), and details of any resulting models** | **Base case or scenario analysis** | **Appraisal committee conclusion** | **Final approach for decision-making** |
| Y |  | Base case | Appraisal committee conclusion | Final approach |
|  |  |  |  |  |
